# Galectin‐3 plasma levels are associated with left atrial contractile function in long‐distance runners

**DOI:** 10.1113/EP093665

**Published:** 2026-07-02

**Authors:** Felipe Contreras‐Briceño, Fernanda Sanhueza‐Olivares, Rodrigo Fernández, Silvana Llevaneras, Pablo F. Castro, Luigi Gabrielli, Mario Chiong

**Affiliations:** ^1^ Instituto de Ciencias de la Salud Universidad de O'Higgins Rancagua Chile; ^2^ Advanced Center for Chronic Diseases, Facultad de Ciencias Químicas y Farmacéuticas Universidad de Chile Santiago Chile; ^3^ Unidad de Investigación Científica Hospital Base San José Osorno Chile; ^4^ Advanced Center for Chronic Diseases Facultad de Medicina Pontificia Universidad Católica de Chile Santiago Chile; ^5^ Laboratorio de Cardiología, Red de Salud UC Christus Pontificia Universidad Católica de Chile Santiago Chile

**Keywords:** atrial strain, biomarker, echocardiography, exercise, heart, myocardial fibrosis, running

## Abstract

High‐intensity endurance exercise induces myocardial remodelling as an adaptive response to sustained volume overload, but excessive cumulative training might also favour deleterious cardiac remodelling, including atrial fibrosis. Whether acute marathon running engages circulating fibrosis‐related biomarkers and whether these biomarkers track with atrial function in a training volume‐dependent manner remains unclear. This study, with within‐subject pre‐ and post‐marathon comparisons, evaluated plasma galectin‐3 and amino‐terminal type III procollagen propeptide (PIIINP) in 36 male long‐distance runners before and immediately after completion of a marathon and examined their relationship to atrial function assessed by speckle‐tracking echocardiography. Participants were stratified into high‐training (HT; ≥100 km week^−1^; *n* = 18) and low‐training (LT; <100 km week^−1^; *n* = 18) groups, with 18 age‐matched non‐active control subjects. At baseline, galectin‐3, PIIINP, left atrial strain a wave (LASa) and right atrial strain a wave (RASa) were comparable across groups. Marathon completion significantly increased galectin‐3 [mean (SD): LT, from 5.2 (2.3) to 23.1 (8.2) ng mL^−1^; HT, from 7.2 (3.8) to 26.5 (11.0) ng mL^−1^; both *P* < 0.001] and PIIINP in both runner groups. The pre‐to‐post fractional changes in galectin‐3 and PIIINP were moderately correlated when all runners were considered together (*r* = 0.569, *P* = 0.006). In exploratory correlation analyses, the post‐marathon galectin‐3 fractional change was positively correlated with the post‐marathon LASa across all runners (*r* = 0.413, *P* = 0.015), indicating that larger increases in galectin‐3 were associated with less negative atrial strain values; subgroup analysis indicated that this association was driven by the highly trained athletes (HT, *r* = 0.476, *P* = 0.045); the corresponding correlation did not reach significance in the LT group (*r* = 0.378, *P* = 0.148). No such association was observed for PIIINP or for the RASa in any group. These findings suggest that the acute galectin‐3 response to marathon running might be linked to left atrial contractile function selectively in highly trained athletes.

## Abstract (highlights)

- **What is the central question of this study?**
  Does marathon running acutely increase circulating fibrosis‐related biomarkers (galectin‐3 and amino‐terminal type III procollagen propeptide) in male long‐distance runners, and is the magnitude of these increases related to atrial contractile function differently in athletes with high versus low weekly training volumes?
- **What is the main finding and its importance?**
  Marathon completion approximately quadrupled plasma galectin‐3 in all runners. The increase in galectin‐3 was correlated with reduced post‐marathon left atrial contractile function, an association driven by the highly trained athletes (≥100 km week^−1^). This training‐dependent finding warrants confirmation in adequately powered prospective studies and might be relevant to the excess atrial fibrillation risk in long‐term endurance athletes.

## 1 INTRODUCTION

Regular physical activity is widely recognised as a cornerstone of cardiovascular health, substantially reducing the risk of coronary heart disease, stroke and chronic heart failure (Lawler et al., 2011; Morris et al., 1953). Current guidelines recommend ≥150 min of moderate or ≥75 min of vigorous aerobic exercise per week for adults (Bull et al., 2020). However, an increasing number of individuals engage in high‐volume intense endurance training, often exceeding 20 h weekly. This sustained volume overload induces structural and functional cardiac adaptations collectively termed ‘athlete's heart’ (D'Andrea et al., 2017).

The athlete's heart phenotype is characterised by biventricular dilatation, increased left ventricular (LV) wall thickness, enhanced trabeculation and modifications in atrial size and function (D'Andrea et al., 2017; Wright et al., 2015). Although these adaptations are generally considered benign and reversible upon detraining (Pelliccia et al., 2010), emerging evidence suggests that continuous vigorous training (37 km week) might offer diminishing mortality benefits and could potentially be deleterious (O'Keefe et al., 2013). This phenomenon, termed ‘Phidippides cardiomyopathy’, describes potential long‐term adverse cardiac effects secondary to repetitive strenuous exercise in predisposed athletes (Trivax & McCullough, 2012). Animal models have demonstrated that long‐term intensive exercise triggers fibrosis in both the atria and the right ventricle, associated with inducible tachyarrhythmias (Benito et al., 2011). In humans, veteran endurance athletes with LV hypertrophy exhibit disrupted collagen turnover, favouring fibrosis (Lindsay & Dunn, 2007), and MRI studies have identified delayed gadolinium enhancement in the interventricular septum of some athletes, indicating myocardial fibrosis (La Gerche et al., 2012). Of particular clinical relevance, high‐intensity endurance training has been associated robustly with an increased risk of atrial fibrillation, plausibly linked to atrial remodelling and fibrosis (Dzeshka et al., 2015; Opondo et al., 2018).

Among biomarkers that capture different stages of the fibrotic cascade, galectin‐3 and the amino‐terminal propeptide of type III procollagen (PIIINP) are particularly attractive (Ionin et al., 2020). Galectin‐3, a β‐galactoside‐binding lectin released by activated macrophages, acts upstream to activate fibroblasts, promote their differentiation into collagen‐producing myofibroblasts and amplify pro‐inflammatory and profibrotic signalling cascades; its rise therefore reflects the initiation phase of fibrogenesis (de Boer et al., 2009; Frangogiannis, 2023). PIIINP, in contrast, is the amino‐terminal propeptide cleaved during the post‐translational assembly of new type III collagen fibrils, and its circulating concentration reflects the downstream, biosynthetic phase of collagen deposition (Ferreira et al., 2017; Zhang et al., 2022). Both biomarkers rise acutely after endurance exercise (Hättasch et al., 2014; Kröpfl et al., 2023; Le Goff et al., 2022; Salvagno et al., 2014), but the relationship between these increases and atrial function, in particular, has been examined inconsistently. Crucially, Hättasch et al. (2014) showed, both in marathon runners and in a murine exercise model, that the postexercise rise in circulating galectin‐3 originates predominantly from skeletal muscle rather than myocardium and identified no clear association with myocardial fibrosis. Whether, despite this contribution of skeletal muscle, a cardiac atrial signal can nevertheless be discerned, and whether this signal depends on cumulative training exposure, is unresolved.

We focused on atrial function for three converging reasons. First, long‐term high‐volume endurance training is among the most consistently demonstrated risk factors for atrial fibrillation in clinical epidemiology (Dzeshka et al., 2015; Opondo et al., 2018). Second, the thin‐walled atria are haemodynamically vulnerable to the repeated volume overload that characterises endurance exercise, and animal models implicate atrial fibrosis as a substrate for arrhythmogenesis in trained hearts (Benito et al., 2011). Third, speckle‐tracking atrial strain is sensitive to early functional change, before chamber dilatation or arrhythmia becomes manifest clinically, and is technically robust in field settings, including the immediate post‐marathon environment, when ventricular strain is often degraded by tachycardia, hyperventilation and motion. Recent multi‐biomarker marathon studies (Lasocka‐Koriat et al., 2025) and ultra‐endurance kinetic studies (Le Goff et al., 2022) have provided valuable insights into the cardiac responses to acute prolonged running but have not coupled galectin‐3 with a sensitive index of atrial contractile function while stratifying a priori by training volume.

The aim of this observational study with within‐subject pre‐ and post‐marathon comparisons was to evaluate plasma levels of galectin‐3 and PIIINP in male long‐distance runners with different training volumes before and immediately after completing a marathon and to examine whether the acute changes in these biomarkers are associated with atrial contractile function assessed by speckle‐tracking echocardiography. We hypothesised that: (1) marathon completion would acutely increase both biomarkers in all runners; and (2) the relationship between acute increases in biomarkers and atrial dysfunction would be more pronounced in highly trained athletes exposed to greater cumulative cardiac stress.

## 2 MATERIALS AND METHODS

### 2.1 Ethical approval

This study adhered to the principles of the *Declaration of Helsinki* and was approved by the Ethics Committee for Human Research at the Faculty of Medicine, Pontificia Universidad Católica de Chile (reference number 160826023). Written informed consent was obtained from all participants prior to any study procedures. The study is reported in accordance with the Strengthening the Reporting of Observational Studies in Epidemiology (STROBE) guidelines where applicable to an observational design with within‐subject pre‐ and post‐marathon comparisons (von Elm et al., 2008); an annotated STROBE checklist is provided as supplementary material.

### 2.2 Study design and participants

A total of 36 male long‐distance runners, aged 18–50 years, were recruited between March and October 2018 for this observational study with within‐subject pre‐ and post‐marathon comparisons. Recruitment was conducted through announcements at local running clubs, social media platforms and direct contact with marathon event organisers in Santiago, Chile. Participants were required to have completed three to five full marathons within the preceding 5 years; first‐time marathon runners were not included, in order to ensure homogeneity of training adaptation. Based on weekly training volume, participants were stratified into two groups: high‐training (HT; ≥100 km week^−1^; *n* = 18) and low‐training (LT; <100 km week^−1^; *n* = 18). The 100 km week^−1^ threshold was selected a priori on the basis of work by Wilhelm et al. (2012), who reported distinct left atrial (LA) remodelling and altered autonomic indices above approximately this lifetime training volume in non‐elite runners, and on broader sports cardiology evidence that adverse atrial remodelling and atrial fibrillation risk become more apparent above this volume (Opondo et al., 2018). Additionally, 18 age‐matched non‐active male subjects without cardiovascular risk factors served as controls (CTR).

Exclusion criteria were as follows: arterial hypertension (resting blood pressure > 140/90 mmHg on two separate occasions), dyslipidaemia (total cholesterol > 200 mg dL^−1^, low‐density lipoprotein > 100 mg dL^−1^, high‐density lipoprotein < 40 mg dL^−1^ or triglycerides > 150 mg dL^−1^), diabetes mellitus, insulin resistance (HOMA index > 2.5), any degree of smoking, cerebrovascular disease history, daily alcohol consumption > 40 g, illicit drug use, nutritional supplement use, impaired kidney function (glomerular filtration rate <60 mL min^−1^ by MDRD equation), family history of sudden cardiac death, liver disease, autoimmune disease, active malignancy, chronic obstructive pulmonary disease, acute inflammation or infection within 1 month prior to the race, and use of antihypertensives, anorectics, antidepressants or antibiotics.

### 2.3 Maximal oxygen consumption

The maximal aerobic capacity was assessed by cardiopulmonary exercise testing (CPET) at the end of the ‘optimal phase’ training period. All runners were instructed not to perform vigorous physical activity during the 48 h before measurement and to avoid intakes of alcohol, caffeine or other stimulants for ≥3 h beforehand. The CPET was performed on a treadmill ergometer (HP Cosmos, Traunstein, Germany) until exhaustion, with verbal encouragement. The protocol has been described in detail previously (Contreras‐Briceño et al., 2021). Cardioventilatory data were analysed breath by breath using open‐circuit spirometry and were expressed under standard temperature, pressure and dry (STPD) conditions (Quark CPET, Cosmed, Rome, Italy). Before each CPET, the gas analyser and the volume transducer were calibrated according to the manufacturer's instructions.

### 2.4 Echocardiographic evaluation

Transthoracic echocardiography was performed on all participants during the week preceding the marathon race, in accordance with American Society of Echocardiography guidelines (Mitchell et al., 2019). Examinations were conducted by a single experienced operator (L.G.), blinded to group allocation, using a Vivid I portable device (GE Healthcare, Horten, Norway) equipped with a 1.5/3.5 MHz cardiac transducer. Standard parasternal and apical views were obtained to assess the geometry and dimensions of the left and right ventricles. LV and atrial volumes were quantified using the two‐dimensional biplane Simpson's method from the apical four‐ and two‐chamber views.

LA and right atrial (RA) dimensions and function were evaluated using strain and strain rate derived from two‐dimensional speckle‐tracking echocardiography (Verdejo et al., 2023). Atrial contractile function was assessed specifically by measuring the negative deformation of the post‐P wave strain curve, reported as left atrial strain a wave (LASa) and right atrial strain a wave (RASa). These values represent the active contraction phase of the atria and are expressed as negative percentages, where more negative values indicate greater contractile function. Post‐marathon echocardiographic acquisitions were obtained within 15 min of race completion. Owing to the physiological conditions of the immediate post‐race setting (tachycardia, hyperventilation and motion), reliable ventricular speckle‐tracking strain analysis could not be guaranteed and was therefore not included in the analysis; LV, LA and RA volumes and atrial strain (post‐P wave segment) met our predefined image‐quality criteria and are reported.

### 2.5 Blood sampling and biomarker analysis

Venous blood samples were collected by trained nurses via direct venepuncture of an antecubital vein into EDTA‐containing tubes. Baseline samples were obtained ∼1 month before the marathon, in standardised conditions. For the pre‐marathon (baseline) blood smapling, participants were instructed to: (1) abstain from vigorous exercise for ≥24 h; (2) abstain from alcohol for ≥48 h; (3) refrain from caffeine intake from the previous evening; (4) sleep for ≥7 h the preceding night; and (5) attend in the morning following an overnight fast of ≥8 h. All participants were non‐smokers, according to the inclusion criteria. The selection of a pre–race window ∼1 month before the marathon coincided with a scheduled instrumented laboratory and echocardiographic visit at the cardiology research unit and the runners’ tapering phase, when training load is reduced and biomarkers should be near their stable trained baseline. Post–marathon samples were collected at the finish line within 15 min of race completion, irrespective of fasting state. All enrolled athletes successfully completed the full 42.2 km marathon distance (*n* = 18 per training group, with no missing biomarker data).

Plasma was separated by centrifugation (3000*g* for 15 min at 4°C) within 30 min of collection and stored at −80°C until analysis. Galectin‐3 and PIIINP concentrations were determined using commercially available enzyme‐linked immunosorbent assay kits (Abcam, Cambridge, MA, USA) according to the manufacturer's protocols. All samples were analysed in duplicate by a single analyst (F.S.‐O.) blinded to group allocation, and mean values were calculated from calibration curves. The intra‐assay coefficients of variation were 8.2% for galectin‐3 and 7.6% for PIIINP.

### 2.6 Sample size considerations

The a priori sample size was estimated using G*Power (v.3.1.9.4) for the within‐group pre‐to‐post change in galectin‐3, based on previous studies in endurance exercise (Hättasch et al., 2014). Using an expected effect size of *f* = 0.40, α = 0.05, power = 0.80 and two groups with two measurements (within‐subject design), the minimum required sample size was estimated at 32 participants; we enrolled 36 runners to allow for potential drop‐outs.

### 2.7 Statistical analysis

Data normality was assessed using the Shapiro–Wilk test. Normally distributed continuous variables are presented as the mean (SD). Between‐group comparisons for baseline characteristics were performed using one‐way ANOVA followed by Tukey's *post hoc* test for multiple comparisons. Pre‐ to post‐marathon changes within and between groups were analysed using a mixed‐effects model, with time (pre vs. post) and group (LT vs. HT) as factors, followed by Fisher's least significant difference *post hoc* test.

Fractional changes in biomarkers were calculated as [(post‐marathon value minus pre‐marathon value)/pre‐marathon value]. Associations between continuous variables were examined using Pearson's correlation coefficient (*r*). Correlation coefficients were interpreted as weak (0.10–0.29), moderate (0.30–0.49) or strong (≥0.50) according to Cohen's guidelines (Cohen, 1988). Effect sizes for ANOVA were reported as partial eta squared (η_p_^2^), with values interpreted as small (0.01–0.06), medium (0.06–0.14) or large (>0.14). Statistical significance was set at *P* < 0.05. All analyses were performed using GraphPad Prism v.9.0 (San Diego, CA, USA). The a priori sample‐size calculation was performed for the within‐group pre‐to‐post biomarker change and not for the subgroup correlations; the correlation analyses by training group should therefore be regarded as exploratory and hypothesis‐generating. Given their exploratory nature and the limited number of prespecified correlation tests, no correction for multiple comparisons was applied to the subgroup correlations, and the corresponding *P*‐values are reported as exact.

## 3 RESULTS

### 3.1 Participant characteristics

All 54 enrolled participants (36 male athletes and 18 male controls; *n* = 18 per group) completed the study with no missing data. Baseline characteristics of the study population are presented in Table 1. Age and height were similar across all three groups. Body weight and body surface area were significantly lower in HT athletes compared with both LT athletes (*P* = 0.032 and *P* = 0.038, respectively) and control subjects (*P* < 0.001 and *P* = 0.004, respectively), consistent with the lean phenotype typically observed in highly trained endurance athletes. The values reported as maximal oxygen consumption were according to the conditions of the athletes [LT, 52.5 (8.1) mL kg^−1^ min^−1^ vs. HT, 58.5 (5.3) mL kg^−1^ min^−1^; *P* = 0.025].

**TABLE 1:** Participant characteristics and pre‐ and post‐marathon echocardiographic and chronotropic measurements

Variable	CTR (*n* = 18)	LT (*n* = 18)	HT (*n* = 18)	CTR versus LT	CTR versus HT	LT versus HT
Age (years)	33.4 (4.6)	38.2 (1.3)	35.8 (6.4)	n.s.	n.s.	n.s.
Weight (kg)	78.8 (8.2)	72.1 (4.5)	65.6 (7.7)	*P* = 0.043	*P* < 0.001	*P* = 0.032
Height (cm)	174.2 (5.9)	175.1 (6.1)	171.8 (7.3)	n.s.	n.s.	n.s.
Body surface area (m^2^)	1.94 (0.12)	1.87 (0.07)	1.78 (0.13)	n.s.	*P* = 0.004	*P* = 0.038
Basal LV diastolic volume (mL)	115.5 (20.2)	122.4 (16.2)	129.2 (22.5)	n.s.	*P* = 0.027	n.s.
Post‐marathon LV diastolic volume (mL)	–	102.5 (21.7)	107.1 (17.3)	–	–	n.s.
Basal LV systolic volume (mL)	47.4 (9.0)	47.8 (10.0)	56.4 (10.2)	n.s.	*P* = 0.014	*P* = 0.018
Post‐marathon LV systolic volume (mL)	–	38.5 (9.2)	43.3 (13.3)	–	–	n.s.
Basal LV stroke volume (mL)	68.1 (12.7)	74.6 (10.4)	72.8 (13.6)	n.s.	n.s.	n.s.
Post‐marathon LV stroke volume (mL)	–	64.0 (14.1)	63.8 (10.9)	–	–	n.s.
Basal LA volume (mL)	35.1 (14.0)	63.7 (20.4)	70.4 (14.9)	*P* < 0.001	*P* < 0.001	n.s.
Post‐marathon LA volume (mL)	–	82.4 (39.7)	79.4 (40.8)	–	–	n.s.
Basal heart rate (beats min^−1^)	69.8 (6.2)	54.6 (4.8)	51.0 (8.0)	*P* < 0.001	*P* < 0.001	n.s.
Post‐marathon heart rate (beats min^−1^)	–	100.6 (9.8)	92.3 (11.5)	–	–	*P* = 0.024
Marathon finishing time (min)	–	222.1 (41.0)	193.0 (31.7)	–	–	*P* = 0.024

*Note*: All values are presented as the mean (SD). Each group comprised *n* = 18 male participants. Between‐group comparisons (CTR vs. LT, CTR vs. HT and LT vs. HT) were performed using one‐way ANOVA with Tukey's *post hoc* test. Pre‐ to post‐marathon changes were assessed using a mixed‐effects model (time × group), followed by Fisher's LSD *post hoc* test. Abbreviations: CTR, control sedentary group; HT, high‐training athletes (≥100 km week^−1^); LA, left atrium; LT, low‐training athletes (<100 km week^−1^); LV, left ventricle.

Echocardiographic findings at rest demonstrated physiological adaptations consistent with athlete's heart. Basal LV diastolic volume was significantly higher in HT athletes compared with control subjects (*P* = 0.027; η_p_^2^ = 0.12, medium effect size), and LV systolic volume was elevated in both athlete groups compared with control subjects. Basal LA volume and resting heart rate differed significantly between athletes and control subjects (*P* < 0.001; η_p_^2^ = 0.45, large effect size), reflecting training‐induced cardiac remodelling and enhanced vagal tone. Marathon completion was followed by a reduction in LV end‐diastolic and end‐systolic volumes in both groups of runners (Table 1), with a small concomitant reduction in calculated stroke volume and a marked rise in heart rate, consistent with the haemodynamic profile of the immediate post‐race state. Marathon completion times were significantly shorter in HT compared with LT runners [193.0 (31.7) vs. 222.1 (41.0) min; *P *= 0.024], demonstrating the superior cardiorespiratory fitness of the HT group.

### 3.2 Biomarkers of myocardial fibrosis

**FIGURE 1:**
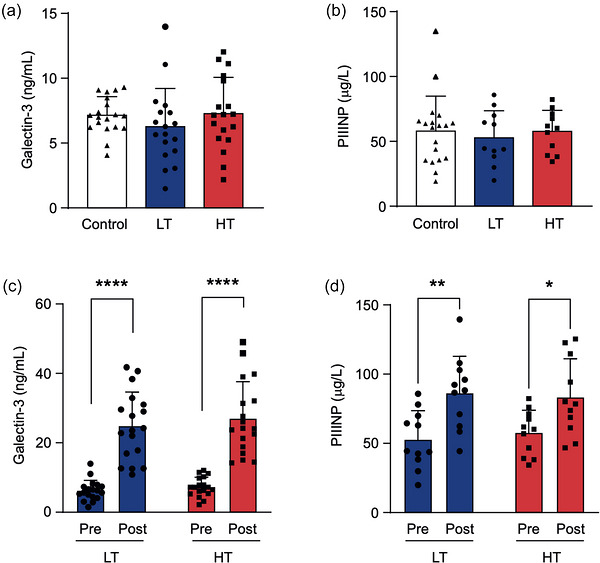
Plasma levels of galectin‐3 and PIIINP before and after marathon running. (a) Baseline galectin‐3 levels in CTR, LT and HT groups. (b) Baseline PIIINP levels across groups. (c) Pre‐ and post‐marathon galectin‐3 levels in LT and HT groups. (d) Pre‐ and post‐marathon PIIINP levels in LT and HT groups. Data are presented as the mean (SD) from *n* = 18 male participants per group. Each runner contributed one paired pre‐ and one paired post‐marathon measurement. Values in (a, b) were analysed using one‐way ANOVA followed by Tukey's *post hoc* test. Values in (c, d) were analysed using a mixed‐effects model followed by Fisher's LSD *post hoc* test. Abbreviations: CTR, control; HT, high‐training; LT, low‐training; PIIINP, amino‐terminal propeptide of type III procollagen.

Baseline plasma levels of galectin‐3 and PIIINP were comparable across CTR, LT and HT groups (Figures 1a, b). Following marathon completion, both biomarkers increased significantly in LT and HT athletes (Figures 1c, d). Galectin‐3 increased from 5.2 (2.3) to 23.1 (8.2) ng mL^−1^ in LT (*P* < 0.001; η_p_^2^ = 0.78, large effect size) and from 7.2 (3.8) to 26.5 (11.0) ng mL^−1^ in HT (*P* < 0.001; η_p_^2^ = 0.72, large effect size). PIIINP increased from 52.8 (18.2) to 85.4 (22.3) µg L^−1^ in LT (*P* = 0.004; η_p_^2^ = 0.35, large effect size) and from 59.1 (15.6) to 82.9 (20.1) µg L^−1^ in HT (*P* = 0.012; η_p_^2^ = 0.28, large effect size). Post‐marathon galectin‐3 and PIIINP levels did not differ between LT and HT groups.

**FIGURE 2:**
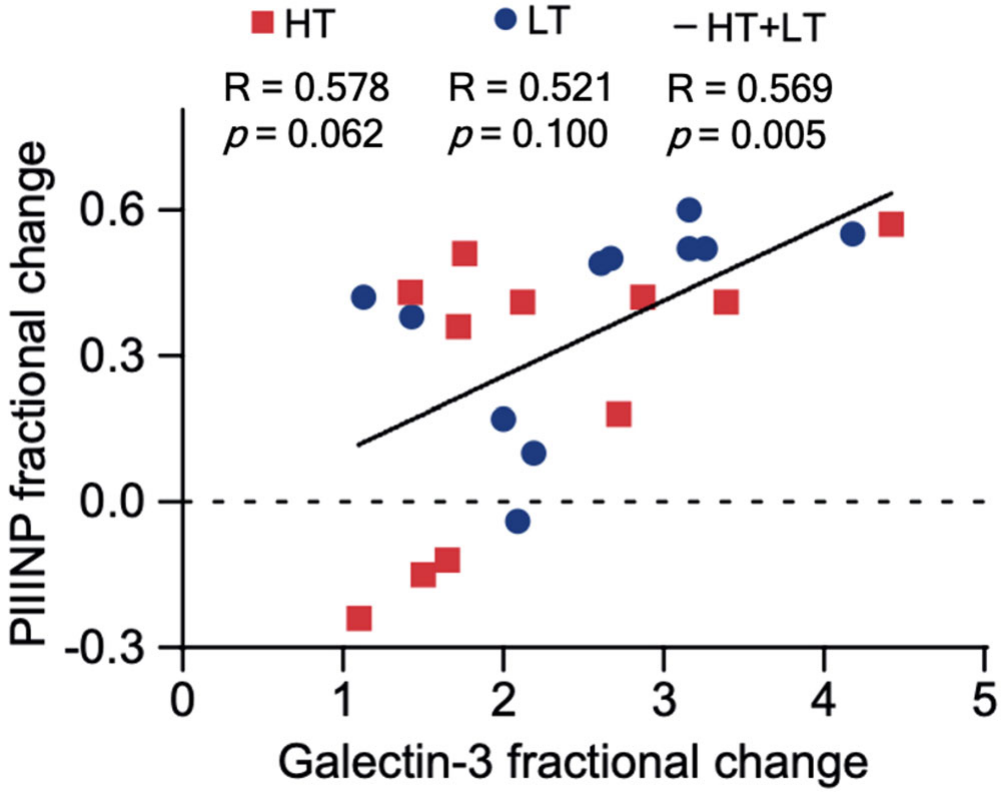
Correlation between fractional changes in galectin‐3 and PIIINP in long‐distance runners. Scatter plot of fractional changes in galectin‐3 and PIIINP for HT (red squares) and LT (blue circles) runners (*n* = 18 per training group; total *n* = 36 for the combined analysis). Pearson correlation coefficients (*r*) and exact *P*‐values are reported for the combined analysis and for each subgroup. Abbreviations: HT, high‐training; LT, low‐training; PIIINP, amino‐terminal propeptide of type III procollagen.

Fractional changes in galectin‐3 and PIIINP demonstrated a moderate positive correlation when all long‐distance runners were considered together (*r* = 0.569, *P* = 0.006; Figure 2). When analysed by training group, this correlation did not reach statistical significance within either group individually (HT, *r* = 0.578, *P* = 0.062; LT, *r* = 0.521, *P* = 0.100), most probably reflecting reduced statistical power in the smaller subgroups.

### 3.3 Atrial function

**FIGURE 3:**
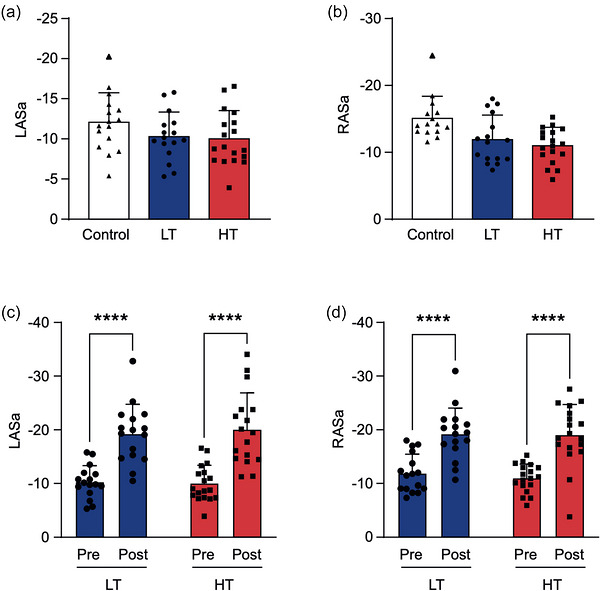
Atrial function measured by LASa and RASa. (a) Baseline LASa values in CTR, LT and HT groups. (b) Baseline RASa values across groups. (c) Pre‐ and post‐marathon LASa in LT and HT groups. (d) Pre‐ and post‐marathon RASa in LT and HT groups. Data are presented as the mean (SD) from *n* = 18 male participants per group. Each runner contributed one paired pre‐ and one paired post‐marathon measurement. More negative values indicate greater atrial contractile function. Values in (a, b) were analysed using one‐way ANOVA followed by Tukey's *post hoc* test. Values in (c, d) were analysed using a mixed‐effects model followed by Fisher's LSD *post hoc* test. Abbreviations: CTR, control; HT, high‐training; LASa, left atrial strain a wave; LT, low‐training; PIIINP, amino‐terminal propeptide of type III procollagen; RASa, right atrial strain a wave.

Baseline LASa and RASa values were similar across CTR, LT and HT groups (Figures 3a, b), indicating comparable atrial contractile function at rest. Post‐marathon, both LASa and RASa increased significantly in magnitude (became more negative) in both LT and HT groups (*P *< 0.001 for all comparisons; η_p_^2^ = 0.65 and 0.58, respectively, both large effect sizes; Figures 3c, d). These changes occurred in the context of reduced LV end‐diastolic and end‐systolic volumes (Table 1) and an elevated heart rate and should therefore not be interpreted as evidence of a primary improvement in atrial function in isolation. The magnitude of these increases was similar between LT and HT athletes.

### 3.4 Association between biomarkers and atrial function

**FIGURE 4:**
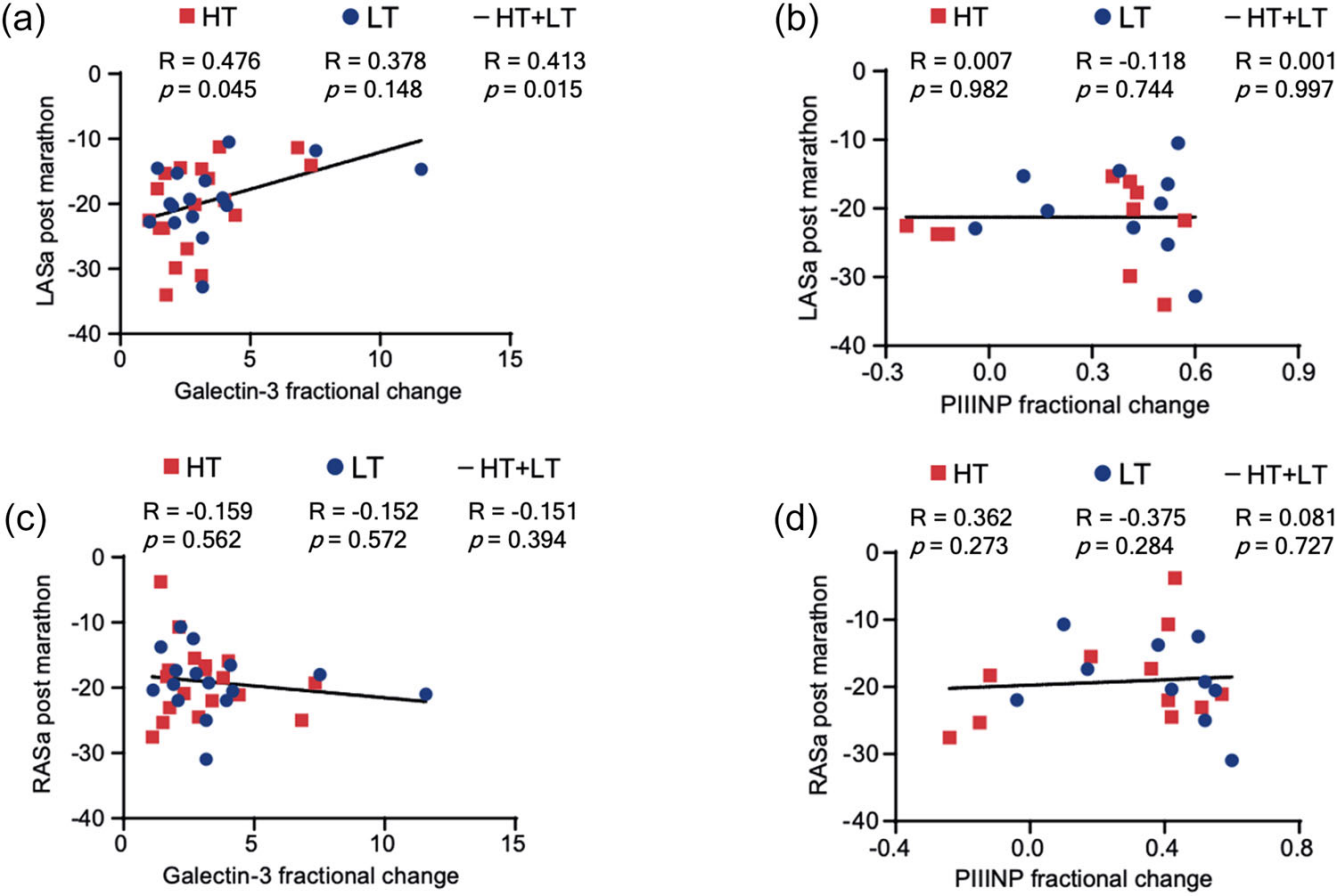
Exploratory correlations between fractional changes in fibrosis‐related biomarkers and post‐marathon atrial function. (a) Correlation between galectin‐3 fractional change and post‐marathon LASa for LT and HT groups. (b) Correlation between PIIINP fractional change and post‐marathon LASa. (c) Correlation between galectin‐3 fractional change and post‐marathon RASa. (d) Correlation between PIIINP fractional change and post‐marathon RASa. Pearson correlation coefficients (*r*) and exact *P*‐values are reported for HT (red squares), LT (blue circles) and combined runners (*n* = 18 per training group; total *n* = 36 for the combined analysis). Abbreviations: HT, high‐training; LASa, left atrial strain a wave; LT, low‐training; PIIINP, amino‐terminal propeptide of type III procollagen; RASa, right atrial strain a wave.

In exploratory correlation analyses, training‐dependent patterns were observed (Figure 4). When considering all athletes together, post‐marathon LASa values showed a significant positive correlation with the galectin‐3 fractional change (*r* = 0.413, *P* = 0.015; Figure 4a). This positive correlation indicates that greater increases in galectin‐3 were associated with less negative (i.e. reduced) LASa values, representing diminished LA contractile function. Subgroup analysis suggested that this association was driven by the HT group, in which the galectin‐3 fractional change was correlated with the post‐marathon LASa (*r* = 0.476, *P* = 0.045); the corresponding association did not reach significance in the LT group (*r* = 0.378, *P* = 0.148). As outlined in the Materials and methods, the a priori sample‐size calculation was performed for the within‐group pre–post biomarker change, not for the subgroup correlations, and these subgroup findings should therefore be regarded as hypothesis‐generating. No significant correlations were observed between PIIINP changes and LASa in either group (Figure 4b). Likewise, neither galectin‐3 nor PIIINP changes were correlated significantly with RASa in either training group (Figure 4c, d).

## 4 DISCUSSION

This study with within‐subject pre‐ and post‐marathon comparisons examined the acute effects of marathon running on plasma fibrosis‐related biomarkers and their relationship to atrial function in long‐distance runners with different training volumes. Our principal findings were 3‐fold: (1) marathon completion acutely increased both galectin‐3 and PIIINP plasma levels regardless of training status; (2) fractional changes in these two biomarkers were moderately correlated, consistent with a linked but mechanistically distinct fibrotic response; and (3) in an exploratory analysis, the acute increase in galectin‐3 was correlated with reduced post‐marathon LA contractile function across all runners considered together (*r* = 0.413, *P* = 0.015), with subgroup analysis indicating that this association was driven by the highly trained athletes (*r* = 0.476, *P* = 0.045) and did not reach significance within the low‐training subgroup, a training‐dependent association we offer as hypothesis‐generating rather than confirmatory.

### 4.1 Acute biomarker responses to marathon running

The significant post‐marathon elevation in galectin‐3 observed in both training groups aligns with previous studies reporting increased galectin‐3 following intense endurance exercise (Hättasch et al., 2014; Kröpfl et al., 2023; Salvagno et al., 2014). The magnitude of the increase in our study (∼4‐fold) is comparable to that reported after 60 km ultramarathons (Salvagno et al., 2014), suggesting that the galectin‐3 response reflects the acute inflammatory and potentially profibrotic stress induced by prolonged high‐intensity exercise. Recent multi‐biomarker work by Lasocka‐Koriat et al. (2025) in 61 amateur marathon runners confirmed a transient post‐race galectin‐3 elevation alongside other cardiac biomarkers and described an inverse correlation between the increase in galectin‐3 and the post‐race reduction in LV ejection fraction, particularly in less‐trained runners (lower maximal oxygen consumption). Le Goff et al. (2022), studying an extreme 330 km mountain ultramarathon, demonstrated biphasic kinetics with several biomarkers, including galectin‐3, reaching their maximum at mid‐race rather than at the finish. Our single immediate post‐marathon sampling is supported for the 42.2 km distance by Hättasch et al. (2014) and Salvagno et al. (2014), but we acknowledge that it might miss an earlier peak.

Likewise, PIIINP increased significantly following the marathon in both groups. PIIINP is released during type III collagen synthesis and serves as a marker of active tissue remodelling (Zhang et al., 2022). Previous studies have documented PIIINP elevations following both single bouts of eccentric exercise (Crameri et al., 2004) and prolonged competitive running (Takala et al., 1986). The moderate correlation between galectin‐3 and changes in PIIINP observed in our study (*r* = 0.569, *P* = 0.006) is consistent with their position along a common pathway but with distinct biological roles, as discussed below.

### 4.2 Skeletal muscle versus myocardial contributions to circulating galectin‐3

A critical interpretive consideration, raised by the seminal work of Hättasch et al. (2014), is that in endurance athletes the postexercise rise in circulating galectin‐3 originates predominantly from skeletal muscle rather than from the myocardium. Hättasch et al. (2014) demonstrated this both in marathon runners, finding no association between the post‐race galectin‐3 rise and indices of myocardial fibrosis assessed by cardiac magnetic resonance, and in a murine exercise model showing that the galectin‐3 increase was attributable mainly to working skeletal muscle. Subsequent work has further established that galectin‐3 is expressed in skeletal muscle and contributes to muscle repair and inflammation. Our data are partly congruent with this skeletal muscle‐dominated framework. We observed a significant positive correlation between the post‐marathon galectin‐3 fractional change and the post‐marathon LASa across all runners considered together (*r* = 0.413, *P* = 0.015; Figure 4a), with subgroup analysis indicating that this association was driven by the HT subgroup (*r* = 0.476, *P* = 0.045) and did not reach significance in the LT subgroup (*r* = 0.378, *P* = 0.148). Three points reconcile this with the work by Hättasch et al. (2014). First, they assessed myocardial fibrosis using late gadolinium enhancement, an index of established structural fibrosis, whereas LA strain a wave is a sensitive index of active atrial contractile function that might reflect more transient, load‐ and remodelling‐related changes not captured by gadolinium enhancement. Second, their cohort was not stratified by long‐term training volume and was smaller than the present combined sample; a training‐dependent signal could plausibly be diluted in an unstratified analysis. Third, and consistent with their main message, our finding does not establish a myocardial source for the circulating galectin‐3 elevation; the association we report is between a (largely skeletal muscle‐derived) circulating signal and an atrial functional index, and is consistent in direction with (but does not prove) a cardiac contribution that becomes discernible in athletes with the greatest cumulative haemodynamic exposure. Parallel responses of skeletal and cardiac tissues to the same prolonged exertion, with the cardiac component becoming detectable only at the higher end of the training–exposure continuum, remain a plausible alternative explanation that future studies with tissue‐specific tracers will need to resolve.

### 4.3 Ventricular–atrial functional coupling and the interpretation of post‐marathon atrial strain

A second interpretive caveat concerns the apparent post‐marathon increase in atrial strain magnitude. Both LASa and RASa became more negative immediately after the race in both groups of runners, consistent on the surface with enhanced atrial contractile function. However, this group‐level change must be considered alongside concomitant changes in ventricular loading. Post‐marathon LV end‐diastolic and end‐systolic volumes were reduced and heart rate was elevated in both runner groups (Table 1), consistent with reduced LV preload and a relative shift of stroke volume contribution towards the late‐diastolic atrial pump. In these exercise stress conditions, an increase in LASa magnitude need not reflect a primary improvement in atrial function and might instead represent compensatory augmentation of the atrial booster pump. This is congruent with the broader strain literature documenting load‐dependent changes in strain indices, and with reports that LV function might itself be transiently reduced post‐marathon (Lasocka‐Koriat et al., 2025). The biologically relevant signal in our data is therefore not the group‐level rise in atrial strain per se, but the training‐dependent association between galectin‐3 rise and reduced LASa within the highly trained group, which is consistent in direction with an adverse rather than purely adaptive atrial response in this subset.

### 4.4 Training volume and atrial vulnerability

The most notable finding of our study was that the association between the increase in galectin‐3 and reduced LA contractile function was observed selectively in highly trained athletes. Three mechanistic considerations are pertinent. First, highly trained athletes experience repeated bouts of atrial stretch and volume overload during prolonged exercise sessions, which might lead to cumulative subclinical atrial remodelling. Animal studies have demonstrated that long‐term intensive exercise induces atrial fibrosis and increases susceptibility to atrial arrhythmias (Benito et al., 2011). In humans, longitudinal studies have shown that high‐intensity endurance training is associated with structural LA changes that might predispose to atrial fibrillation (Dzeshka et al., 2015; Opondo et al., 2018). Second, galectin‐3 has been associated with adverse cardiac remodelling and dysfunction in heart failure populations (Sygitowicz et al., 2021) and with right ventricular dysfunction and exercise intolerance (Zaborska et al., 2020); our findings extend this association to LA function in highly trained athletes. Third, the absence of a similar association with RASa suggests that the left atrium might be more vulnerable than the right atrium to fibrosis‐related dysfunction in this population, possibly because of differences in chamber geometry, wall stress or tissue characteristics, although alternative explanations, including differential sensitivity of the strain measurement, cannot be excluded.

### 4.5 Differential mechanistic roles of galectin‐3 and PIIINP

The absence of a significant correlation between changes in PIIINP and atrial function, despite the postexercise elevation of PIIINP in both groups of runner, deserves a more detailed mechanistic interpretation. Galectin‐3 and PIIINP capture complementary but distinct stages of the fibrotic cascade. Galectin‐3 is an upstream signalling molecule released by activated macrophages that promotes the differentiation of fibroblasts into collagen‐producing myofibroblasts and amplifies transforming growth factor‐β‐ and nuclear factor‐κB‐dependent profibrotic cascades (de Boer et al., 2009; Frangogiannis, 2023); its rise therefore reflects the initiation and signalling phase of fibrogenesis. PIIINP, in contrast, is the amino‐terminal propeptide cleaved from procollagen type III during the post translational assembly of new collagen fibrils, and its circulating concentration reflects the downstream, biosynthetic phase of collagen deposition (Zhang et al., 2022). The moderate correlation that we observed between their post‐marathon fractional changes (*r* = 0.569) is consistent with their position along a common pathway, whereas their divergent behaviour with respect to atrial function is consistent with two further considerations: (1) PIIINP is partly regulated by growth hormone (Equey et al., 2022), which itself increases acutely with intense exercise (Sakharov et al., 2008) and therefore offers a non‐fibrotic source of PIIINP elevation; and (2) PIIINP also reflects extracardiac collagen turnover (skeletal muscle, tendons and skin), which is heavily activated during marathon running, particularly in the eccentric loading phases. Together, these features make PIIINP a less specific reflection of cardiac fibrotic signalling than galectin‐3 in this acute exercise context.

### 4.6 Acute versus chronic galectin‐3 elevations and clinical implications

Several caveats must be emphasised when considering the clinical implications of our findings. First, the post‐marathon galectin‐3 elevation observed here is transient and does not in itself establish pathological fibrosis; it might instead reflect reversible cardiac and skeletal muscle stretch, inflammation and macrophage activation. Second, the clinical evidence linking galectin‐3 to atrial fibrosis, impaired strain and atrial fibrillation is derived chiefly from contexts of chronic, persistent elevation rather than acute postexercise spikes. Third, our protocol included no recovery or long‐term follow‐up samples; whether repeated acute galectin‐3 elevations cumulate, normalise or eventually predict structural or arrhythmic outcomes therefore cannot be determined from these data and must be addressed in prospective longitudinal studies. Given the established link between atrial dysfunction, fibrosis and atrial fibrillation (Dzeshka et al., 2015), the training‐dependent association we describe is biologically plausible as a starting point for hypothesis testing, but our results should not be used to support clinical decisions regarding biomarker‐guided training modification or surveillance in athletes without confirmatory evidence from adequately powered prospective studies.

### 4.7 Limitations

Several limitations should be acknowledged. First, the observational nature of the study and the absence of a recovery or long‐term follow‐up time point preclude inference of causality between elevated galectin‐3 and atrial dysfunction and prevent characterisation of whether repeated acute elevations cumulate to influence long‐term outcomes. Second, the sample was limited to male marathon runners with prior marathon experience; the findings cannot be generalised to female athletes, first‐time marathon runners, other endurance disciplines or older veteran cohorts. Third, the subgroup sizes (*n* = 18 per training group) were modest and the correlation analyses were not the focus of the a priori sample‐size calculation; the principal training‐dependent finding should therefore be regarded as hypothesis‐generating and requiring confirmation in adequately powered prospective studies. Fourth, only a single post‐marathon biomarker sample was obtained. The kinetic studies of Le Goff et al. (2022) in ultra‐endurance settings suggest that biomarker peaks might not coincide with the finish line, and we cannot exclude either earlier or later peaks of galectin‐3 or PIIINP in our participants. Fifth, the baseline blood sample was obtained ∼1 month before the marathon, rather than immediately pre‐race, for logistical reasons related to instrumented laboratory and echocardiographic assessment; any subclinical day‐to‐day variation in biomarker concentrations might have biased the estimates of acute change, and an immediately pre‐race sample would have been preferable. Sixth, ventricular strain and standardised postexercise blood pressure could not be obtained reliably in the immediate post‐race setting and are therefore not reported; this limits direct interpretation of post‐marathon atrial strain as an isolated index of atrial function, as discussed above. Seventh, LV and atrial volumes were obtained by two‐dimensional biplane Simpson's method, which tends to underestimate volumes compared with three‐dimensional echocardiography or cardiac magnetic resonance, particularly in athletic hearts. Eighth, potential confounders, such as diet, hydration status, race‐day environmental conditions (temperature and humidity) and genetic predispositions that could influence biomarker levels and atrial function were not assessed systematically. Ninth, selection bias might exist, because participants were recruited from running clubs and might represent a specific subset of marathon runners. Finally, the 100 km week^−1^ threshold for group stratification, although based on prior literature (Wilhelm et al., 2012), is a pragmatic simplification of a probably continuous dose–response relationship.

Future studies should include larger, more diverse populations of both sexes, multiple time points of biomarker assessment (including intra‐race, immediate post‐race, early recovery and long‐term follow‐up), postexercise functional imaging with rigorous quantification of ventricular and atrial strain alongside loading conditions, and longitudinal follow‐up to determine whether acute biomarker elevations predict long‐term atrial remodelling or clinical outcomes, such as atrial fibrillation.

## 5 CONCLUSION

Marathon completion acutely increases plasma levels of galectin‐3 and PIIINP in male long‐distance runners regardless of training volume. In an exploratory subgroup analysis, only in highly trained athletes was the magnitude of the galectin‐3 increase associated with reduced post‐marathon LA contractile function. Within the limits of an observational study with a single post‐race sampling point and a subgroup correlation analysis for which no formal sample‐size calculation was performed, these findings suggest, as a hypothesis to be tested in adequately powered prospective studies, that the acute galectin‐3 response to marathon running might be linked to LA contractile function selectively in athletes with the greatest cumulative training exposure. They do not, however, establish galectin‐3 as a clinically actionable marker of exercise‐induced atrial dysfunction. Confirmation in prospective studies with serial biomarker sampling, postexercise functional imaging and long‐term follow‐up is required before any clinical implication can be drawn.

## AUTHOR CONTRIBUTIONS

Mario Chiong and Luigi Gabrielli designed the study. Felipe Contreras‐Briceño, Silvana Llevaneras and Rodrigo Fernández organised and performed experiments. Fernanda Sanhueza‐Olivares performed biomarker analyses. Felipe Contreras‐Briceño, Fernanda Sanhueza‐Olivares, Pablo F. Castro, Luigi Gabrielli and Mario Chiong analysed and interpreted the data. Felipe Contreras‐Briceño, Luigi Gabrielli and Mario Chiong drafted and edited the manuscript. All authors approved the final version of the manuscript and agree to be accountable for all aspects of the work in ensuring that questions related to the accuracy or integrity of any part of the work are appropriately investigated and resolved. All persons designated as authors qualify for authorship, and all those who qualify for authorship are listed.

## CONFLICT OF INTEREST

All authors declare no financial or non‐financial competing interests.

## GENERATIVE AI STATEMENT

We utilize artificial intelligence (Claude AI) to enhance editing and improve English language skills.

## Data Availability

Source data for this study are not publicly available owing to privacy restrictions. The source data are available to verified researchers upon reasonable request by contacting the corresponding author.
